# Increased Nutrient Levels Enhance Bacterial Exopolysaccharides Production in the Context of Algae

**DOI:** 10.1111/1758-2229.70071

**Published:** 2025-02-05

**Authors:** Valeria Lipsman, Einat Segev

**Affiliations:** ^1^ Department of Plant and Environmental Sciences Weizmann Institute of Science Rehovot Israel

**Keywords:** algal‐bacterial interaction, marine biofilms, microbial aggregation, nutrient enrichment

## Abstract

Microbial aggregation is central in environmental processes, such as marine snow and harmful marine mucilage events. Nutrient enrichment positively correlates with microbial aggregation. This correlation is largely attributed to the overgrowth of microalgae and the overproduction of agglomerating exopolysaccharides. However, recent studies highlight the significant contribution of bacterial exopolysaccharides to algal‐bacterial aggregation. Here, using controlled laboratory experiments and environmental metatranscriptomic analysis, we investigate the impact of nutrient enrichment on bacterial exopolysaccharides production, while bacteria are in the context of their algal hosts. Our findings demonstrate a marked increase in bacterial exopolysaccharides production in response to a relative increase of inorganic phosphorus and nitrogen levels, both in the lab and in the environment. These results highlight the interplay between nutrient regimes, bacterial physiology and microbial aggregation in marine ecosystems and emphasise gaps in our understanding regarding the bacterial role in environmental processes that involve microbial aggregation.

## Graphical abstract

This study examined the effect of inorganic phosphorus and nitrogen enrichment on marine bacteria in the context of their algal hosts (I), Both bacteria and algae show an increase in exopolysaccharides production (II), These exopolysaccharides can promote algal‐bacterial agglomeration (III) and potentially play a role in environmental microbial aggregation processes.

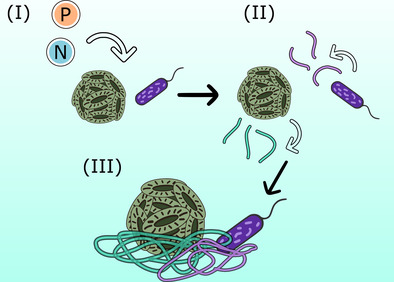

## 1 Introduction

Marine ecosystems are profoundly influenced by microbial communities, which play essential roles in nutrient cycling and primary production (Field et al. 1998). Microbes are key in the functioning of the biological carbon pump as they facilitate the transfer of carbon from the surface ocean to the deep sea (Ploug and Grossart 2000). Marine snow—comprised of organic and inorganic materials, including phytoplankton, faecal pellets and microbial exopolymers—serves as a significant mediator of this carbon transport (Turner 2015; Simon et al. 2002). The microbial communities within marine snow are highly active, driving the decomposition and remineralization of organic matter (Ploug and Grossart 2000). Additionally, microbial aggregation processes can lead to harmful events such as marine mucilage, which involves the accumulation of gelatinous organic matter in coastal waters (Danovaro, Fonda Umani, and Pusceddu 2009). These mucilage events can adversely affect marine ecosystems by causing oxygen depletion, smothering benthic organisms and disrupting aquaculture activities (Danovaro, Fonda Umani, and Pusceddu 2009). Understanding the role of microbial exopolysaccharides (EPS) in these aggregation processes is important for comprehending their impact on marine ecosystems (Danovaro, Fonda Umani, and Pusceddu 2009).

A positive correlation exists between elevated nutrient levels and enhanced microbial aggregation, both in terrestrial and marine ecosystems. The increased availability of nutrients appears to stimulate microbial growth, biofilm formation and the subsequent aggregation of microorganisms, leading to the formation and stabilisation of soil and marine aggregates (Wilpiszeski et al. 2019; Baragi and Anil 2016; Chen et al. 2022). In marine microbial communities, the synergistic effect of elevated temperature, pCO_2_ and nutrients has a significant impact on biofilm formation and community composition. This highlights the role of nutrient enrichment in enhancing microbial aggregation in marine environments, and indeed harmful mucilage events were found to develop under conditions of increased nutrient levels (Savun‐hekimoğlu and Gazioğlu 2021; Malone and Newton 2020).

Recently, we demonstrated that *Phaeobacter inhibens* bacteria contribute a specific EPS that promotes the formation of an algal‐bacterial extracellular matrix (ECM) and enhances aggregation in co‐culture with *Emiliania huxleyi* algae (Lipsman et al. 2024). Building upon these findings, we sought to investigate whether elevated nutrient levels influence the bacterial contribution to EPS production. To address this question, we first examined the influence of elevated nutrient level on EPS production in controlled laboratory model systems. We simulated elevated nutrient regimes by manipulating the concentrations of inorganic phosphorus and nitrogen levels in the growth media of algal‐bacterial co‐cultures. By separately quantifying the bacterial and algal EPS, we observed a marked increase in both algal and bacterial EPS production in response to high levels of inorganic phosphorus and nitrogen. To gain insights into the ecological relevance of our observations, we conducted a metatranscriptomic analysis of natural bacterial communities that were sampled during an algal bloom induced by an influx of inorganic nutrients. Consistent with our laboratory results, the metatranscriptomic data revealed elevated expression of bacterial genes involved in EPS biosynthesis following the influx of inorganic nutrients. Furthermore, elevated expression of bacterial EPS genes correlated with the detection of various EPSs. These findings collectively support that bacteria exhibit increased EPS production following an influx of elevated nutrient levels, suggesting a bacterial contribution to the EPS pool under nutrient enriched conditions in marine environments. Our study highlights the interplay between nutrient levels, bacterial physiology and microbial aggregation in marine ecosystems.

## 2 Results

### 2.1 Elevated Levels of Phosphate and Nitrate Increase Algal and Bacterial EPS Production

**FIGURE 1:**
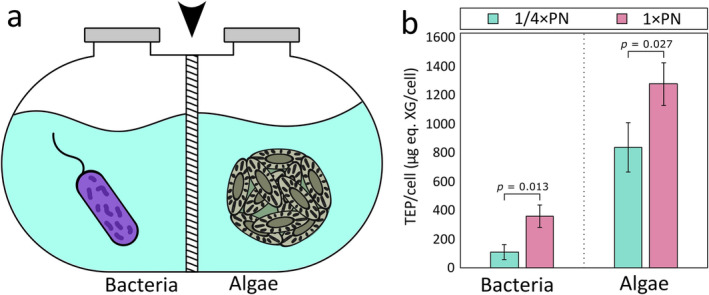
Elevated concentrations of phosphate and nitrate promote algal and bacterial EPS production. (a) A schematic representation of the dual‐cultivation chambers, showing bacteria (left chamber) and algae (right chamber) separated by a membrane (marked by an arrowhead). (b) Bacterial (left) and algal (right) cultures were treated with ¼ × PN (teal) or 1 × PN (pink) nutrient regimes. EPS was quantified at early stationary phase (10 days) by measuring absorbance at 787 nm of AB bound to TEP and converting to Xanthan Gum (XG) equivalent weight. TEP/cell was calculated by dividing total TEP by the corresponding cell numbers (see Section 4). Statistical significance was assessed using a one‐tailed 2‐sample *t*‐test with three biological repeats, with *p*‐values indicated above each organism.

We wished to assess the effect of inorganic phosphate (P) and nitrate (N) on EPS production of *P. inhibens* bacteria and *E. huxleyi* algae. Evaluating the impact of nutrient levels on bacteria and algae, separately, is challenging when cultivating co‐cultures of this algal‐bacterial pair. To address this challenge, we utilised specialised dual‐cultivation chambers equipped with a 0.22 μm pore size membrane (Figure 1a). This membrane separates algae and bacteria, allowing only small molecules to pass through. Consequently, the membrane maintains a shared chemical environment between the chambers while ensuring the separation of macromolecules that are produced by either algae or bacteria, such as EPS. Similar cultivation experiments have been previously conducted demonstrating that membrane‐separated dual‐cultivation chambers allow successful microbial metabolic exchange (Thøgersen et al. 2018; Vallet et al. 2019). Algal and bacterial EPS production was tested under two P and N regimes. First, we added the recommended concentrations of nutrients to the L1‐Si growth medium (Guillard and Hargraves 1993), containing 36 μM P and 882 μM N (we denote this nutrient regime as 1 × PN). These nutrient levels induce bloom‐like growth of both algae and bacteria, resulting in cell densities of ~10^6^–10^7^ cells/mL each (Lipsman et al. 2024). Similar P and N concentrations have been shown to increase algal EPS production, with levels five times higher than those in f/10 medium, the lower‐nutrient precursor of L1 medium (Buzzelli et al. 1997). Additionally, a threefold increase in P and N concentrations has been observed to trigger algal blooms in natural environments, which was followed by a significant rise in EPS abundance (Sidhu et al. 2023). Thus, we supplied a quarter of the L1 P and N concentrations (denoted as ¼ × PN), containing 9 μM P and 220 μM N and compared them to the 1 × PN regime, which served as a control for nutrient concentrations that induce *E. huxleyi* EPS production. At the early stationary phase of algal growth, cultures from each side of the dual‐cultivation chambers were harvested. EPS production was assessed by the quantification of Alcian Blue (AB), a dye that binds to acidic EPS named Transparent Exopolymeric Particles (TEP), known to be produced by both algal and bacterial species (Passow 2002).

Our data show that higher P and N concentrations affect both algal and bacterial EPS production (Figure 1b). *E. huxleyi* TEP production was significantly higher (*p* = 0.027) under the 1 × PN nutrient regime, compared to the ¼ × PN regime, as previously demonstrated for other algal species (Buzzelli et al. 1997). Under both nutrient regimes, algae produce more TEP per cell than bacteria. However, bacteria also showed significantly higher (*p* = 0.013) TEP production under higher P and N concentrations, highlighting the potential bacterial contribution to aggregation at sea in response to elevated nutrient levels.

### 2.2 Marine Bacteria Express EPS‐Related Genes in Response to a Phosphate and Nitrate Influx

After observing increased bacterial EPS levels in response to elevated P and N concentrations in laboratory experiments, we aimed to examine if a similar bacterial response occurs in the environment. Recently, a study following the metatranscriptomics of bacterial populations during an algal bloom reported a peak of P and N environmental levels (Supplementary Figure 1), prompting a secondary algal bloom with elevated levels of EPS (Supplementary Figure 2) (Sidhu et al. 2023). The increase in EPS was mainly attributed to algal production. We examined the publicly available metagenomics and metatranscriptomics data throughout the period of elevated EPS to assess whether upregulation of bacterial genes that are associated with EPS biosynthesis occurs after the peak of P and N.

**FIGURE 2:**
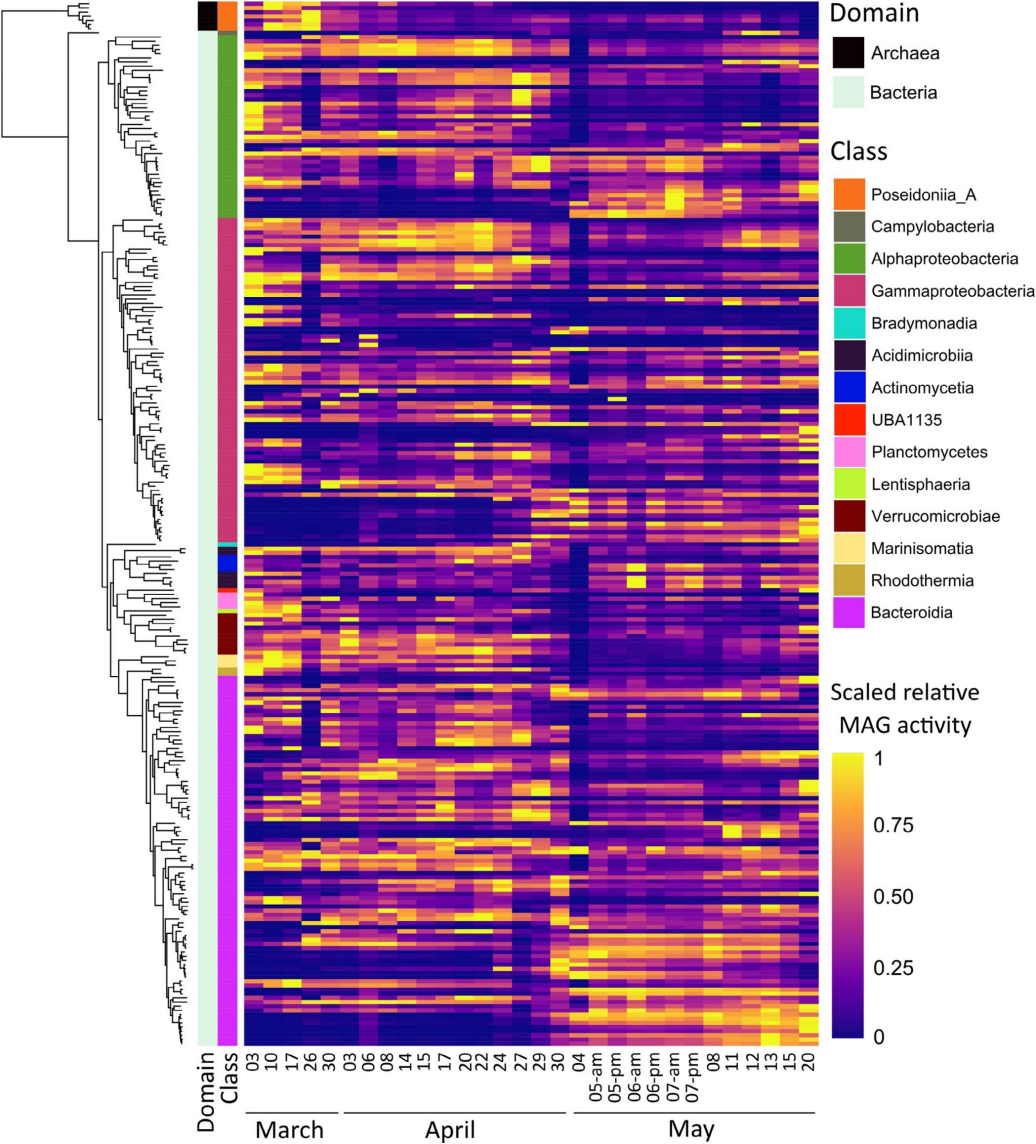
A peak of phosphate and nitrate increases the relative activity of specific bacterial groups in the environment. Phylogenetic tree of the 244 bacterial and 7 archeal representative MAGs (created by GTDB‐Tk (Chaumeil et al. 2020) with domain and class affiliations (left). The relative activity (scaled) of each MAG is represented as a heatmap (right). Relative activity was calculated by summing the TPM values of all ORFs mapped to a MAG and dividing it by the sum of the TPM of all ORFs in a sample. This value was further normalised by the proportion of the MAG length compared to the total lengths of all MAGs. Data adapted from Sidhu et al. (2023).

From the collected metagenomic data, 251 representative metagenomic assembled genomes (MAGs) were previously generated and published (Sidhu et al. 2023). Of these 251 MAGs, 7 MAGs belonged to Archaea and the rest were bacterial MAGs. To achieve a general view of the bacteria that are present in the analysed samples, we constructed a phylogenetic tree of the MAGs (Figure 2). The primary bacterial classes represented by the MAGs belonged to *Alphaproteobacteria*, *Gammaproteobacteria* and *Bacteroidia*. To assess the relative activity of the MAGs along the duration of the analysis, the transcriptomic data was mapped onto the representative MAGs and the counts were normalised by transcript per million (TPM). Relative activity was determined by mapping the relative TPM values to a certain MAG and normalising to the relative size of the MAG. During the sampling period, several MAGs exhibited higher activity during the second bloom that was induced by a nutrient influx in comparison to the initial natural bloom (Supplementary Figure 1). These active MAGs belonged to the *Alphaproteobacteria* and *Bacteroidia* classes.

To follow the expression of genes related to EPS production in the MAGs, we focused our analysis on bacterial genes involved in alginate, cellulose and wzx/wzy‐dependent EPS biosynthesis (Vandana and Das 2022). These gene modules are involved in the production of many characterised bacterial EPSs, including acidic polysaccharides which potentially contribute to the TEP pool (Schmid, Sieber, and Rehm 2015). Additionally, we have previously shown that some of these bacterial modules are expressed by bacteria in co‐culture with algae, contributing to algal‐bacterial aggregation (Lipsman et al. 2024). To characterise EPS biosynthesis modules in the representative MAGs, we searched for gene clusters of the key functions in this biosynthetic pathway (Supplementary Table 1).

**FIGURE 3:**
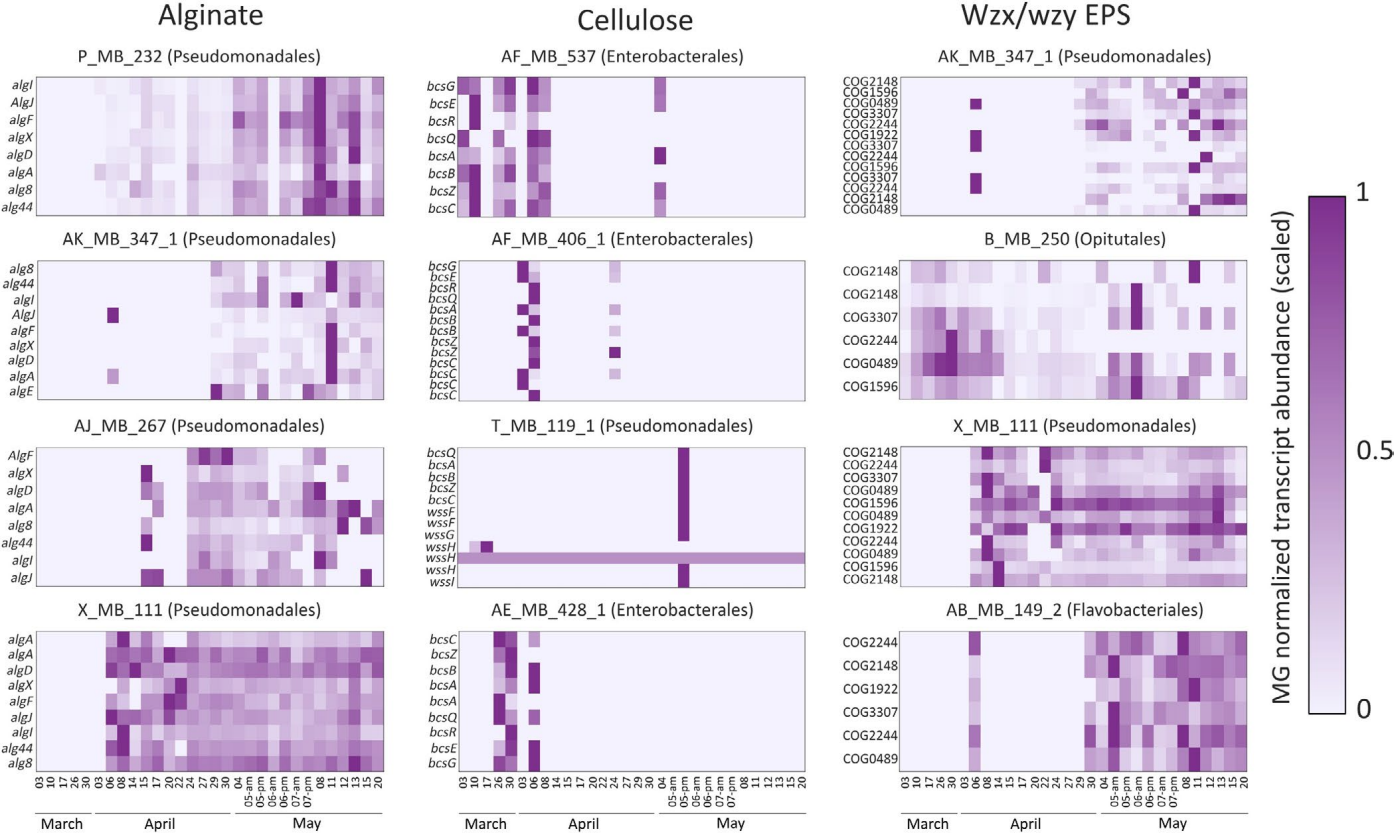
A peak of phosphate and nitrate increases the expression of bacterial genes related to EPS biosynthesis in the environment. The expression profiles of the gene clusters potentially involved in the biosynthesis of specific EPS during the two algal bloom phases. Expression is presented as the scaled transcript abundance normalised by the abundance of 10 marker genes (MG) of each MAG. These values serve as the number of transcripts of a specific gene per MAG (Salazar et al. 2019) (see Section 4). Gene clusters related to the biosynthesis of alginate, cellulose and wzx/wzy dependent EPS are shown for different MAGs. Shown are the genes (for alginate and cellulose) or the OG numbers associated with the wzx/wzy‐dependent EPS biosynthesis genes (annotated using EggNOG‐mapper v2 (Cantalapiedra et al. 2021). Above each gene cluster is the MAG name and the order annotated for that MAG. Data adapted from Sidhu et al. (2023).

We identified several MAGs that harbour gene clusters that putatively encode most of the biosynthesis capacity of the different EPSs (Figure 3). Several alginate and wzx/wzy‐dependent gene modules exhibited higher expression during the second nutrient‐induced algal bloom compared to the initial natural bloom phase. In contrast, the cellulose gene modules exhibited higher expression only at a single time point following the nutrient influx. Antibody‐based measurements conducted by Sidhu et al. (2023) demonstrated increased abundance of various EPSs from the 5th to 7th of May following the nutrient influx (Supplementary Figure 2). Notably, on May 5th and 6th, elevated expression was observed for genes belonging to all of the three types of EPS modules—alginate, cellulose and wzx/wzy‐dependent EPS. Given that bacteria are capable of producing diverse EPSs, with some resembling algal EPSs such as alginate and cellulose (Vandana and Das 2022), it cannot be ruled out that the EPSs that were previously identified are of both algal and bacterial origin. The co‐occurrence of elevated expression of bacterial EPS modules and the increased EPS abundance suggest that bacteria may contribute to EPS production in response to P and N influx in the environment.

## 3 Discussion

In this study, we demonstrate that bacterial EPS production increases following a nutrient influx in both laboratory settings and marine environments. Our results demonstrate that higher concentrations of P and N lead to increased EPS production in both *E. huxleyi* algae and *P. inhibens* bacteria. Specifically, *E. huxleyi* algae showed a significant increase in TEP production under the higher nutrient regime compared to the lower nutrient regime. This observation aligns with previous studies that demonstrated nutrient‐induced EPS production in algal species (Buzzelli et al. 1997). Notably, while algae produced more TEP per cell than bacteria, bacteria also showed a significant increase in TEP production under elevated nutrient conditions. It is important to note that the nutrient concentrations used in our laboratory experiments are higher than those typically found in natural environments. While the fold‐changes in nutrient levels under both laboratory and environmental nutrient conditions are similar, the absolute nutrient levels tested in the laboratory are not directly comparable to those in natural settings. Although we observed significant changes in EPS production at higher nutrient levels, these conditions may not fully reflect the nutrient limitations experienced by bacteria in natural environments. Importantly, the interaction between algae and heterotrophic bacteria, particularly in nutrient‐limited environments, plays a significant role in shaping microbial communities. Algae release molecules that influence bacterial physiology, and heterotrophic bacteria depend on algae to release exudates and transform inorganic compounds like nitrite into utilisable forms (Segev et al. 2016). Therefore, elevated nutrient levels may have a direct impact on bacterial EPS metabolism or an indirect effect through the algal host.

Our metagenomic and metatranscriptomic analyses of a marine algal bloom revealed that several bacterial MAGs exhibited higher activity during the secondary nutrient‐induced bloom phase compared to the initial natural bloom. The previously published data that was analysed here showed that the composition and abundance of both algal and bacterial species varied significantly between the two algal bloom phases (Sidhu et al. 2023). It was previously suggested that the availability of algal polysaccharides can impact the structure of bacterial communities. The algal polysaccharides provide essential nutrients for heterotrophic bacteria and can form a dense matrix that facilitates bacterial attachment and aggregation (Bar‐Zeev et al. 2012). In turn, bacteria in proximity to their algal hosts can contribute to the EPS pool, promoting further algal‐bacterial agglomeration (Lipsman et al. 2024). The elevated expression of bacterial EPS modules in environmental samples suggests a potential bacterial contribution to marine aggregation processes specifically in nutrient‐rich environments. Our understanding of algal‐bacterial aggregation in the environment is partial at best. The current study highlights the complex dynamics between algal and bacterial communities and emphasises the potential impact of nutrient influxes on the ecology of microbial populations.

Influxes of waters that carry enriched N and P levels can impact the composition and spatial traits of various aquatic microbial populations (Yi, Lo and Cheng 2020; Wang et al. 2023). Eutrophication, the increase in nutrient levels, was reported to encourage growth of harmful species, change trophic networks, and alter the diversity and richness of the ecosystem (Dubey and Dutta 2020). Elevated levels of phosphate and nitrogen in marine environments are known to play a significant role in promoting marine mucilage events (Volkan Oral 2023). Marine mucilage can carry distinct bacterial communities (Danovaro, Fonda Umani, and Pusceddu 2009) which can play a role in these events by producing EPS and further contributing to mucilage formation. These mucus layers can have profound ecological impacts, including the disruption of marine food webs, the suffocation of marine organisms and the alteration of biogeochemical cycles (Danovaro, Fonda Umani, and Pusceddu 2009). Understanding the bacterial role in these events is crucial for developing strategies to manage and mitigate their occurrence in marine ecosystems.

## 4 Materials and Methods

### 4.1 Microbial Strains and Growth Conditions

The bacterial strain *Phaeobacter inhibens* DSM 17395 was acquired from the German Collection of Microorganisms and Cell Cultures (DSMZ, Braunschweig, Germany). Bacteria were cultivated by inoculation from a frozen stock (−80°C) onto ½ YTSS agar plates containing 2 g yeast extract, 1.25 g tryptone and 20 g sea salt per litre (all obtained from Sigma‐Aldrich, St. Louis, MO, USA). Plates were incubated at 30°C for 2 days. Individual colonies were used to initiate bacterial cultures in artificial seawater (ASW) medium. Bacterial starters were supplemented with CNPS to support bacterial growth. CNPS consisted of L1‐Si medium (detailed below) (Segev et al. 2016; Zech et al. 2009) supplemented with glucose (5.5 mM), Na_2_SO_4_ (33 mM), NH_4_Cl (5 mM) and KH_2_PO_4_ (2 mM), all sourced from Sigma‐Aldrich. The cultures were incubated at 30°C with continuous shaking at 130 rpm for 2 days until the start of the experiments (day 0).

The axenic algal strain *Emiliania huxleyi* CCMP3266 was obtained from the National Center for Marine Algae and Microbiota (Bigelow Laboratory for Ocean Sciences, Maine, USA). Algae were cultivated in L1 medium based on the protocol by Guillard and Hargraves (1993). Notably, Na_2_SiO_3_ was omitted as per cultivation recommendations for this algal strain, and the medium was designated as L1‐Si. Algal cultures were maintained in standing conditions within a growth room at a temperature of 18°C under a light/dark cycle of 16/8 h. Light intensity during the illuminated period was maintained at 150 mmoles/m^2^/s. The absence of bacteria in axenic algal cultures was periodically verified through plating on ½ YTSS plates and microscopic observations.

### 4.2 Microbial Growth in Dual‐Cultivation Chambers

Dual‐cultivation chambers were custom made based on Vallet et al. (2019) (Bein M.LTD, Tel Aviv, Israel). A 100 mm 0.22 μm GV Durapore membrane filter (from Sigma‐Aldrich) divided between chambers in the dual‐cultivation setup. Each side of the split chamber contained 30 mL of medium with the regular concentrations of vitamins and trace metals of L1‐Si medium. Phosphate (NaH_2_PO_4_∙H_2_O, denoted as P) and nitrate (NaNO_3_, denoted as N) were either supplied in one‐fourth of the regular L1‐Si (denoted as ¼ × PN) or regular L1‐Si concentrations (denoted as 1 × PN).

Algal cell concentrations from a late exponential phase culture were enumerated using a CellStream CS‐100496 flow cytometer (Merck, Darmstadt, Germany) with 561 nm excitation and 702 nm emission. Each sample involved recording 50,000 events. An inoculum of 10^4^ algal cells was introduced into one side of the split chamber. *P. inhibens* bacteria (prepared as previously described) were quantified by measuring OD_600_ values using an Ultrospec 2100 pro spectrophotometer (Biochrom, Cambridge, UK) with plastic cuvettes. Cell numbers were adjusted based on a pre‐existing calibration curve to inoculate a concentration of 10^2^ cells/mL in 30 mL of medium, resulting in 10–100 colony forming units (CFU) per mL, which were verified by plating serial dilutions (in ASW) on ½ YTSS plates. Bacteria were inoculated into one side of the split chambers following 4 days of algal growth in the other side. Following bacterial inoculation, the split chambers were incubated for 10 additional days under the same conditions as described above for algal cultivation.

Bacterial concentrations in split chambers were determined by counting CFU/mL. The cultures were serially diluted in ASW and plated on ½ YTSS plates. The plates were incubated at 30°C for 2 days, and subsequently, colonies were enumerated to calculate bacterial concentration in CFU/mL. Algal cell concentrations were quantified using a flow cytometer, as previously described. Cultures were thoroughly mixed and subsequently, 100 μL of the samples were transferred to round‐bottom 96‐well plates. Each sample was recorded for 1 min to capture a total of > 50,000 events. Cross‐contamination was examined by monitoring the CFU/mL and flow cytometry samples of both algal and bacterial sides at the time of initial inoculation and sample collection.

### 4.3 TEP Measurement

Alcian Blue (AB) staining was performed following published protocols (Passow 2002) using a staining solution with 200 mg L^−1^ of AB (8GX, Sigma‐Aldrich). The solution was prepared in acidified ultrapure water, adjusting the pH to 2.5 with glacial acetic acid. Each culture was filtered on Isopore 25 mm 0.4 μm PC membrane (Sigma‐Aldrich) in three biological repeats, with 6 and 2 mL of culture for bacteria and algae, respectively. Following filtration, the membranes were washed twice with 1 mL of DDW and stained with 1 mL AB for 10 s. The dye was removed, and the filters were washed twice with 1 mL of DDW. Xanthan Gum (XG, Sigma‐Aldrich) was used for calibration by dissolving 75 mg XG in 1 L DDW, mixing for 3 h. Serial dilutions where prepared by mixing 0.125, 0.250, 0.500, 0.750 and 1 mL of the XG with DDW to a final volume of 1 mL. The dilutions were then mixed with 0.5 mL AB, filtered and washed as described above. The stained filters were transferred to 15 mL falcon tubes with 80% sulfuric acid and mixed for 2 h. Absorbance was measured at 787 nm wavelength and TEP_0.4μm_ concentrations were calculated as described in Equation (1) (Passow 2002):

(1) $${\mathrm{TEP}}_{0.4\mathrm{\mu m}}=\frac{\left(A_{\text{sample}}-A_{\text{filter blank}}\right)}{m_{787}\times V}$$

where TEP_0.4μm_ is expressed as XG μg/μL. m787 is the slope of the calibration curve of XG. *V* is the volume of sample filtered. *A*_sample_ is the absorbance of AB extracted from a sample filter. *A*_filter blank_ is the absorbance of AB extracted from a filter used with only medium. As blank, 80% sulfuric acid was used. Each result was divided by the corresponding cells/μL or CFU/μL values to calculate TEP_0.4μm_/cell values.

### 4.4 MAG Analysis

A collection of 251 MAGs was previously assembled by Sidhu et al. (2023) and re‐analysed in the current study. MAG phylogenetic affiliations were re‐assigned using GTDB‐Tk v2.3.2 (Chaumeil et al. 2020) with GTDB v214. A phylogenetic tree was constructed using de_novo_wf command of GTDB‐Tk v2.3.2 (Chaumeil et al. 2020). The tree was extracted and visualised using the ape and ggtree packages in R v4.3.1. ORFs prediction was conducted using Prodigal (Hyatt et al. 2010). The ORFs were annotated using EggNOG‐mapper v2 (Cantalapiedra et al. 2021). HMM homology was searched with HMMER3 (Eddy 2009) against the Pfam‐A HMM database (Finn et al. 2016). HMM models of biosynthetic genes related to EPS biosynthesis were acquired from the KEGG KofamKOALA (Aramaki et al. 2020) and EggNOG v5.0 (Huerta‐Cepas et al. 2019) databases. The hmm models were used to search for orthologs in the representative MAGs with HMMER3 (Eddy 2009). Manual annotation of the biosynthetic EPS modules was conducted by searching gene clusters that contain the ORFs associated with the key genes of the core functions of the biosynthetic pathways (Vandana and Das 2022), summarised in Supplementary Table 1.

### 4.5 Metatranscriptomics Analysis

Raw sequence data was acquired for 30 time points from the European Nucleotide Archive (accession PRJEB52999) (Sidhu et al. 2023). Ribosomal RNA reads were removed using SortMeRNA v4.3.6 (Kopylova, Noé, and Touzet 2012) searched against the smr_v4.3_default_db database. Messenger RNA reads were quality trimmed and end repaired using the bbduk and repair scripts from the BBMap v39.01 (https://sourceforge.net/projects/bbmap/). Reads with a minimum length of 70 bp were then mapped to all 251 representative MAGs using Bowtie2 v2.5.1 (Langmead and Salzberg 2012). Counts were calculated by FeatureCounts (Liao, Smyth, and Shi 2014). First, a transcript per million (TPM) normalisation was conducted for all samples as described in Equation (2) (Wagner, Kin, and Lynch 2012).

(2) TPM=Reads mapped toORFLength ofORFinkbp∑Reads mapped toORFLength ofORFinkbp×106

To assess MAG activity, the sums of TPM values of each MAG in a given sample were normalised as described in Equation (3) (Mickol et al. 2021):

(3) MAGactivity=ΣTPMvalues ofallORFs mapped to theMAGΣTPMvalues ofallORFs inasampleΣcontig lengths of theMAGinkbpΣlengths of contigs ofallthe MAGs inasampleinkb

For expression analysis of polysaccharide‐related genes in a MAG, the counts were first transformed with marker genes (MG) normalisation, as described previously (Salazar et al. 2019), using 10 universal single‐copy phylogenetic MGs as OGs (Sunagawa et al. 2013; Milanese et al. 2019): COG0012, COG0016, COG0018, COG0172, COG0215, COG0495, COG0525, COG0533, COG0541 and COG0552. Genes were extracted using FetchMGs v1.2 (available at http://motu‐tool.org/fetchMG.html). The normalisation was conducted as described in Equation (4) (Salazar et al. 2019):

(4) MGTranscript Abundancei=reads mapped to anORFORFlengthinkbmedian10MGsfromagenome106

As the MGs were shown to be constitutively expressed across many different conditions (Milanese et al. 2019), MGs normalised transcript abundance can be interpreted as the relative per‐MAG number of transcripts of a given ORF (Salazar et al. 2019). MAGs containing clusters of genes related to core functions, which are highly conserved, were selected and transcript abundance was plotted with MATLAB R2023b.

## Author Contributions

**Valeria Lipsman:** conceptualization, investigation, writing – original draft, visualization, formal analysis, writing – review and editing. **Einat Segev:** conceptualization, funding acquisition, writing – original draft, writing – review and editing, supervision.

## Conflicts of Interest

The authors declare no conflicts of interest.

## Supporting information


**Data S1.** Supporting Information.

## Data Availability

Data sharing not applicable to this article as no datasets were generated or analysed during the current study.
