# Supplementary material for: Increased Nutrient Levels Enhance Bacterial Exopolysaccharides Production in the Context of Algae
Source: Environ Microbiol Rep. 2025 Feb 5;17(1):e70071. doi: 10.1111/1758-2229.70071 (PMC11799575; doi:10.1111/1758-2229.70071)
Supplement: Supplementary file 1 — Data S1. Supporting Information. [file EMI4-17-e70071-s001.pdf]

Supplementary Data

Supplementary Table 1. The core functions of the genes in biosynthesis of different EPSs, with KEGG, OG and main Pfam ID domains

EPS	Gene	KEGG	OG	PFAM
Alginate	alg8	K19290	COG1215	
	alg44	K19291		PilZ
	algK	K19292	COG0790	Sel1
	algX	K19293		ALGX
	algI	K19294	COG1696	MBOAT
	algJ	K19295		ALGX
	algF	K19296		AlgF
	algE	K16081		Alginate_exp
	algG	K01795	COG3420	Beta_helix
	algL	K01729		Alginate_lyase
Cellulose	bcsA	K00694	COG1215	Cellulose_synt
	bcsB	K20541	COG1215	BcsB
	bcsZ	K20542	COG3405	Glyco_hydro_8
	bcsC	K20543	COG5010	BCSC_C
	bcsG		COG2194	CBP_BcsG
	bcsE			CBP_BcsE
	bcsR			CBP_BcsR
	bcsQ			CBP_BcsQ
Wzx/Wzy	Primary glycosyl transferase		COG2148	Bac_transf
	Repeating unit flippase		COG2244	Polysacc_synt
	Repeating unit polymerase		COG3307	Wzy_C
	Polysaccharide co-polymerase		COG0489	Wzz
	Outer membrane polysaccharide export		COG1596	Poly_export
	Repeating unit tranferase		COG1922	Glyco_tran_WecG

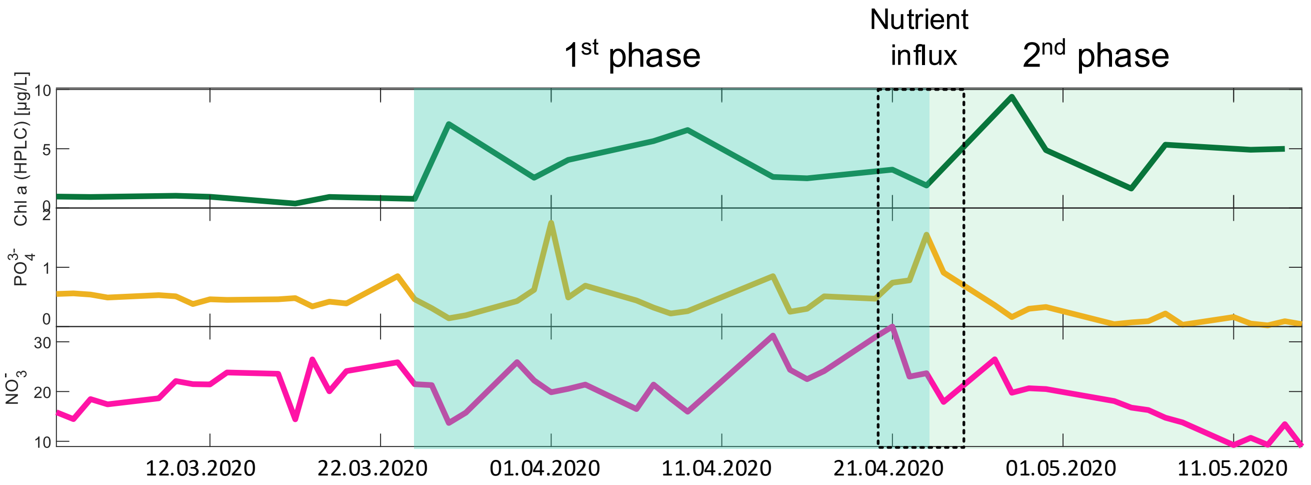

Supplementary Figure 1. Environmental data during the sampling period. Measurements of Chlorophyll *a* (top), NO_3^- (bottom) and PO_4^{3-} (middle) concentrations during the period of the analysis. Marked are the two algal bloom phases and the time of the nutrient influx. Data adapted from Sidhu, *et. al.*, 2023[1].

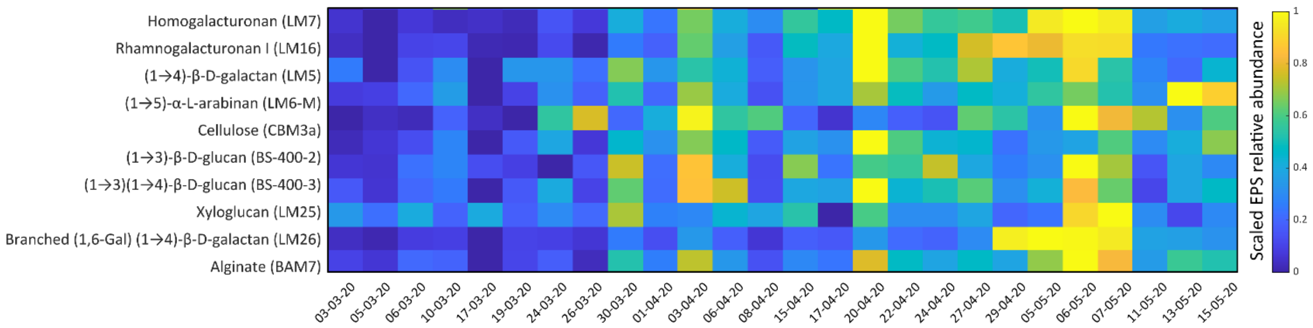

Supplementary Figure 2. Abundance of specific EPSs during the sampling period. Antibody-based measurements of different dissolved EPSs during the period of the analysis. Shown are various EPS and the monoclonal antibody used to target the EPS. EPS was extracted from the high molecular weight dissolved organic matter (HMWDOM) of each sample, using EDTA as solvent. Values indicate antibody signal intensity. Color bar shows values that were scaled for each EPS and are presented as the fraction of the highest measured abundance. Data adapted from Sidhu, *et. al.*, 2023[1].

1. Sidhu, C. *et al.* Dissolved storage glycans shaped the community composition of abundant bacterioplankton clades during a North Sea spring phytoplankton bloom. *Microbiome* **11**, (2023).
